# Comparative assessment of Oral Hygiene and Periodontal status among children who have Poliomyelitis at Udaipur city, Rajasthan, India

**DOI:** 10.4317/medoral.17658

**Published:** 2012-05-01

**Authors:** Mridula Tak, Ramesh Nagarajappa, Archana Sharda, Kailash Asawa, Aniruddh Tak, Sagar Jalihal

**Affiliations:** 1 MDS, Post graduate student, Dept. of public health dentistry, Pacific dental college and hospital, Debari, Udaipur, Rajasthan, India; 2 MDS, Professor and Head of Department, Dept. of public health dentistry, Pacific dental college and hospital, Debari, Udaipur, Rajasthan, India; 3 MDS, Senior lecturer, Dept. of public health dentistry, Pacific dental college and hospital, Debari, Udaipur, Rajasthan, India; 4 MDS, Post graduate student, Dept. of Oral Pathology and Microbiology, Pacific dental college and hospital, Debari, Udaipur, Rajasthan, India

## Abstract

Objective: To assess and compare the oral hygiene and periodontal status among children with Poliomyelitis having upper limb disability, lower limb disability and both upper and lower disability at Udaipur city, Rajasthan, India. Study design: Total sample comprised of 344 Poliomyelitis children (upper limb disability: 33.4%; lower limb disability: 33.7%; both upper and lower limb disability: 32.9%) in the age group of 12-15 years. Clinical examination included recording Simplified Oral Hygiene Index and Community Periodontal Index. Analysis of variance (ANOVA), multiple logistic and stepwise linear regression were used for statistical analysis. Results: The mean OHI-S (2.52±1.05) score was found to be highest among children who had both upper and lower limb disability (p<0.05). The highest and lowest mean number of healthy sextants were found among those with only lower limb disability (4.53±2.05) and among those with both upper and lower limb disability (0.77±1.39), respectively (p<0.05). Stepwise multiple linear and multiple logistic regression analysis showed that the best predictor for oral hygiene and periodontal status was limb involved in the disability. Conclusion: The results of the study depicted an overall poor oral hygiene and periodontal status of the group. It was recognized that limbs involved in the disability had an impact on the oral hygiene and periodontal condition. The situation in this specialized population draws immediate attention for an integrated approach in improving the oral health and focus towards extensive research.

** Key words:**Poliomyelitis, upper limb disability, lower limb disability, oral hygiene, periodontal status.

## Introduction

Disability has often been described as a physiological deficit. Current concepts of disability, however, are based on social models, which describe disability in terms of functional limitations experienced by a person because of environmental and social barriers. More specifically, a person with a disability has been defined as anyone who has or has had an impairment causing a long term adverse effect upon his or her ability to perform daily activities typical for the person’s stage of development and cultural environment (1). Disability affects a wide segment of population of all ages and social classes (2). According to World Health Organization, an estimated 650 million people live with disabilities around the world (3) and census 2001 has revealed that over 21 million people in India are suffering from one or the other kind of disability. This is equivalent to 2.1% of the population (4).

Poliomyelitis (polio) is a highly infectious viral disease caused by a virus. The virus enters the body through the mouth and multiplies in the intestine. Initial symptoms are fever, fatigue, headache, vomiting, stiffness in the neck and pain in the limbs. One in 200 infections leads to irreversible paralysis. It can strike at any age but mainly affects children under five years of age. Polio cases have decreased by over 99% since 1988, from an estimated 350 000 cases then, to 1997 reported cases in 2006. The reduction is the result of the global effort to eradicate the disease. In 2008, only four countries in the world remain polio-endemic, down from more than 125 in 1988. The remaining countries are Afghanistan, India, Nigeria and Pakistan (5).

Till date, we found no studies pertaining to the comparison of oral health according to limbs involved in the disability among children with Poliomyelitis. But there are other studies which signify the effect of functional disability on oral health. Walton et al. (6) in his review on oral health and Juvenile idiopathic arthritis demonstrated a detrimental effect of Juvenile Idiopathic arthritis on oral health. In a study by Pischon N et al. (7), subjects with Rheumatoid arthritis had significantly increased periodontal attachment loss. Poor periodontal status had also been found in Chinese teenagers with cerebral palsy in a previous study (8). Impaired mobility and ability to reach services may be the factors which affect the uptake of dental care. Problems with physical access to health service premises and dental surgeries are reported. These problems may be more profound in rural areas where accessibility, availability and affordability of health services may be the issues of concern. Upper limb disability may affect the individual’s ability to manage effective oral hygiene (2). In many instances, the oral hygiene care of a child with disability becomes the responsibility of another person, generally a parent or guardian. Because oral hygiene affects one’s esthe-tics and communication, it has strong biological, psychological, and social projections (9). People with disabilities deserve the same opportunities for oral health and hygiene as those who are healthy. Unfortunately, oral health care is one of the greatest unattended health needs of the people with disabilities (2).

Although, the evidence has demonstrated that, as a group, persons with disabilities have more untreated periodontal disease and a poorer state of oral hygiene than the general population (10); there is still a paucity of literature on oral health status of population with disability, particularly for physical disability. So this study has been undertaken with the objectives to assess and compare the oral hygiene and periodontal status among children with Poliomyelitis having upper limb disability, lower limb disability and both upper and lower disability at Udaipur city, Rajasthan, India.

## Material and Methods

-Study area: Udaipur city is situated in the southern part of Rajasthan state located in western India. It is bordered on the north by Rajsamand district, on the south by Dungarpur and Banswara, on the east by Chittorgarh and on the west by Pali and Sirohi districts of Rajasthan and Sabar Kantha district of Gujarat state. According to census 2001, the population of Udaipur city was 3,89,438 (11).

The study was conducted in a special care hospital (Narayan Sewa Sansthan) and all special care hostels of Udaipur city. The Narayan Sewa Sansthan is the only special care hospital at Udaipur city. According to hospital records, the total number of Poliomyelitis children (12-15 years age group) who visited in the previous year was found to be 1,466.

-Study Design: A cross sectional descriptive survey was conducted among the 12-15 years old children with Poliomyelitis visiting the special care hospital (Narayan Sewa Sansthan) and residing in all special care hostels during August to September 2010. The sample size thus obtained was 344.

-Ethical approval - Before the commencement of the study, ethical approval was obtained from the ethical committee of the Pacific Dental College and Hospital, Udaipur, Rajasthan.

Informed consent: Written informed consent was obtained from the authorities of the hospital and hostels and also from the guardians of the patients at the hospital.

-Proforma details: The first part of the proforma consisted of collection of details regarding demographic information, place, socioeconomic status, limbs involved in disability, oral hygiene practices, consumption of snacks in between meals and previous visit to the dentists. This information was recorded by the investigator herself. Socioeconomic status was recorded according to Prasad’s Classification of socioeconomic status scale (12) based on which it was stratified into 5 categories, viz, Upper High, High, Upper Middle, Lower Middle and Poor. With regard to place of residence, children were categorized into 2 groups: urban and rural.

The second part of the proforma consisted of the indices to be recorded, namely Oral Hygiene Index- Simplified (13) and Community Periodontal Index (14). A brief description of both the indices is as follows:

1. Oral Hygiene Index- Simplified (OHI-S): This index which is meant to assess the oral hygiene status has two components, the Debris Index-Simplified (DI-S) and the Calculus Index-Simplified (CI-S) which are calculated separately and are summed up to get Oral Hygiene Index-Simplified for an individual. The examination is done using mouth mirror and explorer. The interpretation of index is as follows: Good- 0 to 1.2, Fair- 1.3 to 3.0, Poor- 3.1 to 6.0 (13).

2. Community periodontal Index (P): This index includes examination of index teeth in each sextant for three indicators of periodontal status, viz, gingival bleeding, calculus and periodontal pockets. Clinical examination is performed using mouth mirror and CPI probe (14).

Independent variables for regression analysis included gender, place, socioeconomic status, limbs involved in disability, oral hygiene practices, consumption of snacks in between meals, previous visit to the dentists and oral hygiene status.

For the purpose of multiple logistic regression analysis, some variables were dichotomized as follows:

1. Socioeconomic status: High and Low. The Upper high and High Socioeconomic status group were merged together to form High socioeconomic status group and the other three (Upper middle, lower middle and poor) were merged together to form Low socioeconomic status group.

2. Limbs involved in disability: Upper limb disability and Lower limb disability. The group having both upper and lower limb disability was merged with group having upper limb disability.

3. Oral Hygiene Practices: Those using tooth brush and tooth paste; and those using other oral hygiene aids (Finger, Neem twig, tooth powder and others).

4. Previous visit to the dentists: Regularly and Irregularly. Those who visited dentist regularly every 6 months were grouped as “visiting regularly” and those who had never visited a dentist and visits only when there is a problem were grouped as “visiting irregularly”.

5. Oral Hygiene status: Good and Poor. Fair oral hygiene status group was merged with group having poor oral hygiene status.

Before the commencement of the study, training and intraexaminer calibration was done in the Department of Public Health Dentistry, Pacific Dental College and Hospital (kappa value for OHI-S= 0.88, kappa value for CPI = 0.86).

-Pilot Survey: A pilot survey was conducted among 25 subjects at the special care hospital to assess the validity and accuracy of the proforma and to know the practical and communication difficulties while examining oral cavity of this group of subjects.

-Methodology: In accordance with the schedule, the investigator visited the special care hospital and hostels. The purpose of the study was informed and explained to participants and their guardians and after obtaining the consent, all subjects were interviewed to obtain information in the questionnaire and were examined clinically. Clinical oral examination was carried out in the wards of special care hospital and in the well lighted rooms in special care hostels under adequate natural light using mouth mirror, explorer and CPI probe (American Dental Association Type III examination) (15) in accordance with World Health Organization criteria and methods (9).

-Statistical analysis

Data was analyzed using the Statistical Package for Social Sciences version 15.0 software. ANOVA, stepwise linear multiple regression and multiple logistic regression were used for comparisons. p- value of less than 0.05 was considered as statistically significant.

## Results

 Table 1 shows distribution of study subjects according to age, gender, place, socioeconomic status, oral hygiene practices, consumption of snacks in between meals, previous visits to dentists and limb involved in disability/handicap. Among the total 344 participants, 115 had upper limb disability, 116 had lower limb disability and 113 had both upper and lower limb disability. There were 174(50.6%) males and 170(49.4%) females.

**Table 1:**
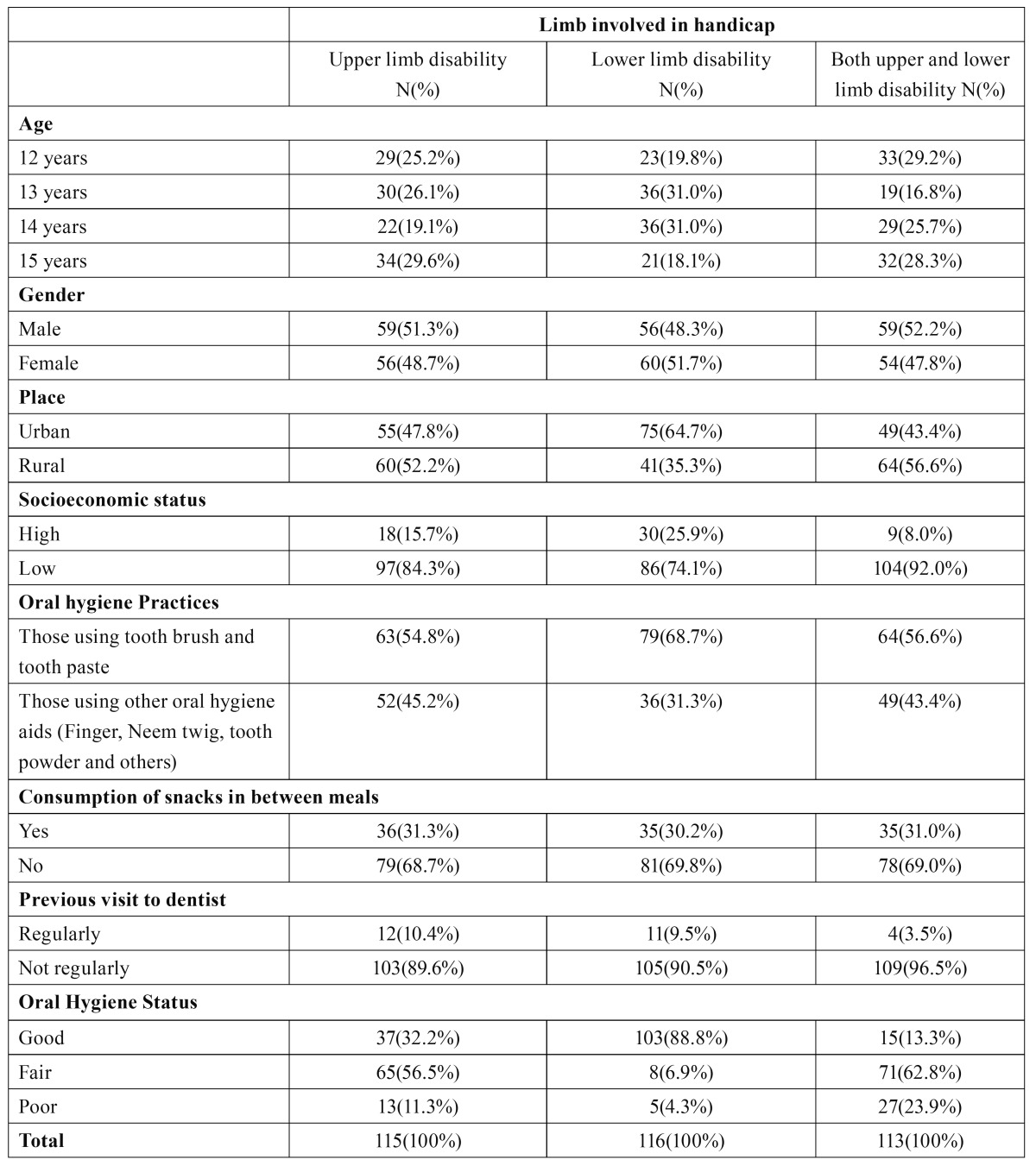
Distribution of study subjects.

 Table 2 shows mean Debris Index- Simplified, Calculus Index- Simplified and Oral Hygiene Index- Simplified according to limb involved in the disability. Total mean OHI-S score was 1.72 ± 1.22. Mean DI-S, CI-S and OHI-S were significantly greatest in subjects with both upper and lower limb disability, followed by those with only upper limb disability and lowest among those with only lower limb disability. Mean OHI-S score for subjects with both upper and lower limb disability, only upper limb disability and only lower limb disability were 1.88 ± 1.06, 0.79 ± 0.84 and 2.52 ± 1.05, respectively.

**Table 2:**
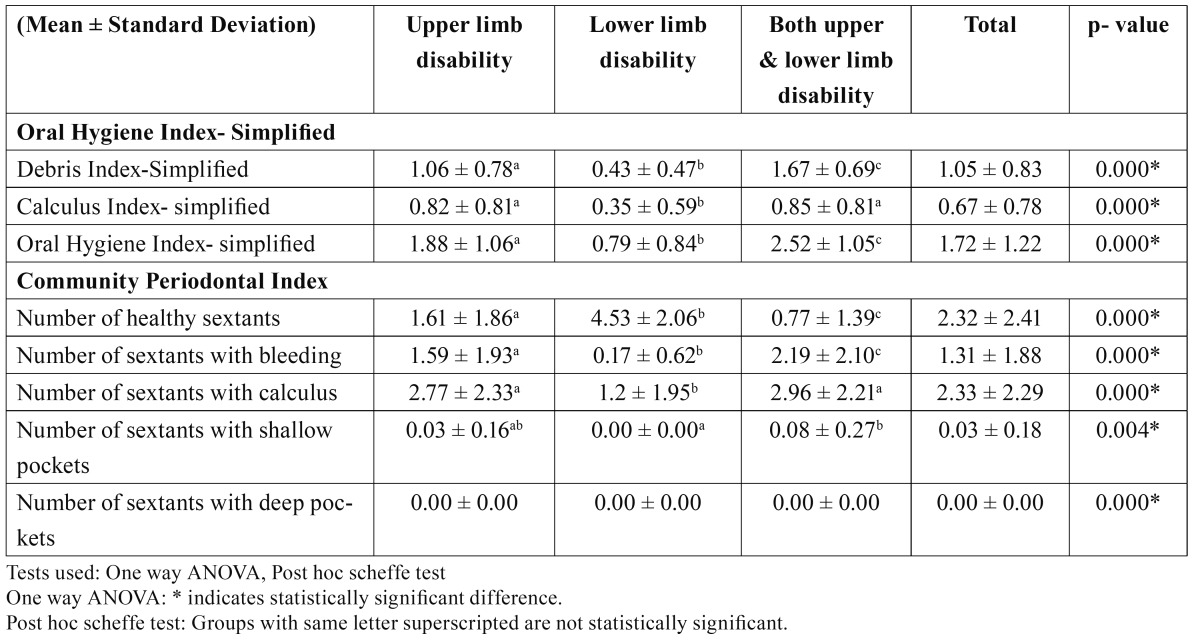
Mean Debris Index - Simplified, Calculus Index - Simplified and Oral Hygiene Index – Simplified and number of sextants affected by periodontal disease according to limb involved in disability.

Mean number of healthy sextants were significantly highest among the subjects with only lower limb disability (4.53 ± 2.06) followed by those with only upper limb disability (1.61 ± 1.86) and lowest among those with both upper and lower limb disability (0.77 ± 1.39). There was a significant difference in the mean number of sextants with bleeding between the three groups, the highest being in the subjects with both upper and lower limb disability (2.19 ± 2.10), followed by subjects with only upper limb disability (1.59 ± 1.93) and then with only lower limb disability (0.17 ± 0.62). Mean number of sextants with calculus differed significantly between the subjects with only lower limb disability (1.29 ± 1.95) and the other two groups, that is, subjects with only upper limb disability (2.77 ± 2.33) and both upper and lower limb disability (2.96 ± 2.21) but the difference was not significant between the subjects with only upper limb disability and both upper and lower limb disability. Mean number of sextants with shallow pockets was found to be 0.03 ± 0.18 and the difference was only significant between lower limb disability group and both upper and lower limb disability group. No deep pockets were found among all the three groups.

 Table 3 shows Stepwise multiple linear regression analysis which was executed to estimate the linear relationship between OHI-S and CPI as dependent variables and various independent variables. The best predictors in the descending order for OHI-S were limb involved in the disability, place and socioeconomic status. The amount of variance obtained for limb involved in the disability, place and socioeconomic status were 39.1%, 49.2%, 50.4%, respectively. The best predictors in the descending order for CPI were limb involved in the disa-bility and oral hygiene status. The amount of variance obtained for limb involved in the disability and oral hygiene status were 36.3% and 39.7%, respectively.

**Table 3:**
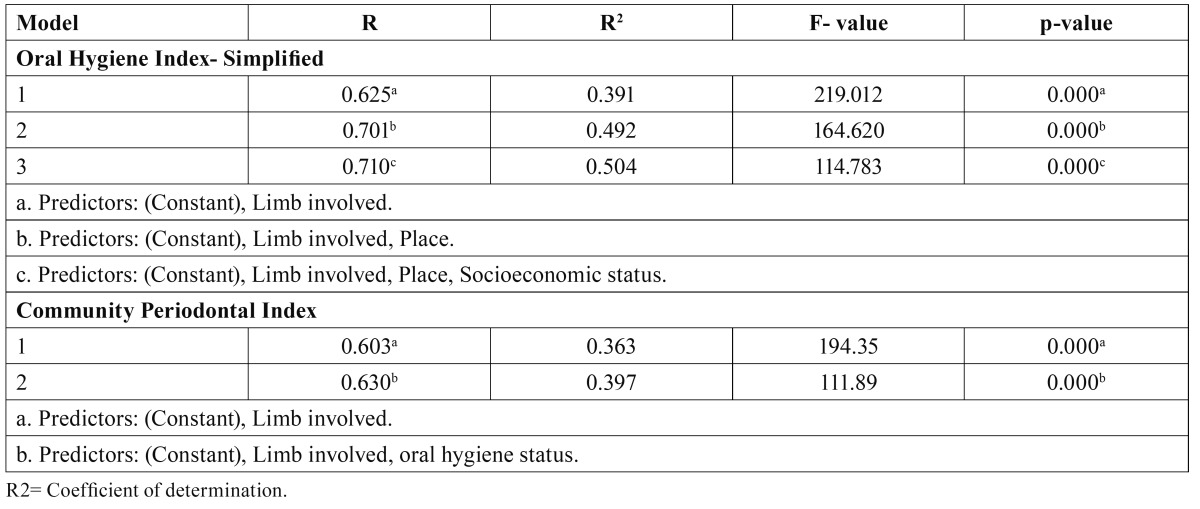
Stepwise multiple linear regression analysis with Oral Hygiene Index- Simplified and Community Periodontal Index as dependent variables.

 Table 4 reveals that odds ratio for poor oral hygiene status was significantly higher among subjects with upper limb disability as compared to those with lower limb disability, among subjects residing in rural areas than among those residing in urban areas and among those with low socioeconomic status than among those with high socioeconomic status. This table also shows that odds ratios for periodontal disease were significantly higher among upper limb disabled subjects than lower limb disabled subjects and among those who were having good oral hygiene practices and status.

**Table 4:**
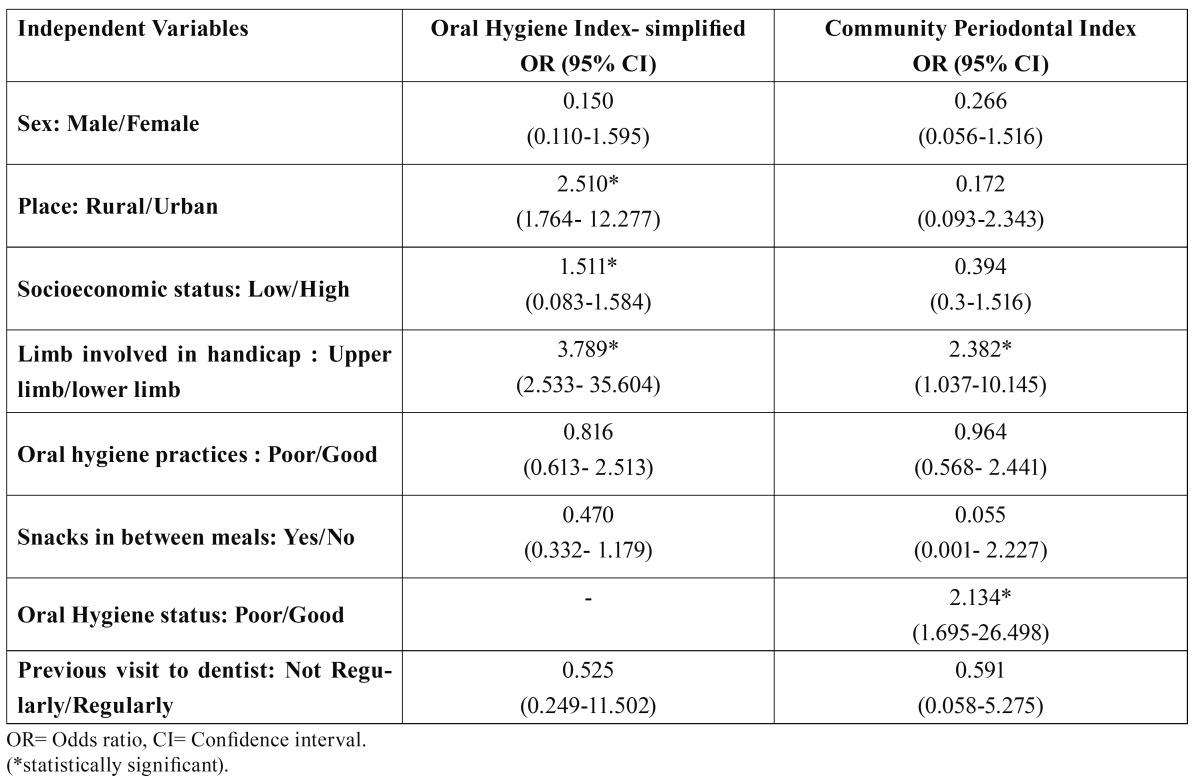
Odds ratio (OR) and 95% confidence interval (CI) for oral hygiene and periodontal status according to multiple logistic regression.

## Discussion

We know that people with a physical disability are entitled to equal standards of health and care as their able bodied cohorts (2) and dental care is one of the top unmet health needs of persons with special needs (16). There is evidence that they experience poorer oral health (14,17) and barriers to achieving good oral health and accessing appropriate dental services (18-20). Brown and Schodel reviewed 32 studies of children with disability and reported that such patients tend to have poorer oral hygiene than their non disabled counterparts. Most of these findings highlight the difficulties encountered by individuals with disability in maintaining an adequate level of oral hygiene (21). Majority of the studies conducted on special needs population has provided data regarding the oral hygiene and periodontal status of various disability groups or comparison with normal population. This cross sectional study assess and compares the oral hygiene and periodontal status among the children with Poliomyelitis having upper limb disability, lower limb disability and both upper and lower limb disability. The findings depict poor oral hygiene and periodontal status of the group. The most obvious reason may be their physical inability to clean the oral cavity adequately (22).

The findings of the study indicated that majority of the subjects with upper limb disability (83.5%) and those with both upper and lower limb disability (85.8%) were unable to brush their teeth themselves. This shows their dependence for maintaining oral hygiene either on parents or caretaker. As we know that physical handicap or limb disability certainly hampers the day to day activities of an individual, so when we talk about oral hygiene maintenance which is an important aspect of daily life and routine of an individual, it is more compromised if upper limb is disabled. The participants visiting dentist regularly every 6 or 12 months were only 37(10.8%) which is lesser than the findings of Gizani et al. (23) who reported 49.3% of 12 year old children with disabilities attending dentist regularly. The lesser number in lower limb disability group may be attributed to the impaired mobility and ability to reach services. If this group be transported for care, a little higher commitment to oral hygiene may be expected from them.

-Oral Hygiene Status:

Good oral hygiene was exhibited by 45.05% of subjects while there were 41.9% and 13.1% subjects with fair and poor oral hygiene, respectively which was worse than that of a previous study conducted on children with disabilities attending a day institution at Lagos (9). The prevalence of good oral hygiene status among subjects with lower limb disability was highest (88.8%), followed by subjects with only upper limb disability (32.2%) and those with both upper and lower limb disability (13.3%). The overall mean OHI-S score was found to be 1.72 ± 1.2 which was higher than the normal school children of the same age group (15), the individuals with disability in the age range of 3- 26 years (9) and in the age range of 12-15 years (24). The mean DI-S, CI-S and OHI-S of subjects with lower limb disability were significantly lower than the other two groups. This may be due to the inefficiency or dependence of subjects with upper limb disability for oral hygiene maintenance. The difference in mean DI-S and OHI-S was also significant between upper limb disability group and subjects with both upper and lower limb disability. This may be attributed to the fact that disability of both the limbs renders them more dependent. The results of stepwise multiple linear regression and multiple logistic regression depicted a poor oral hygiene in upper limb disability group (Odds Ratio = 3.789, 95% CI= 2.533- 35.604), those residing in rural areas (Odds Ratio = 2.510, 95% CI= 1.764 - 12.277) and with low socioeconomic status (Odds Ratio = 1.511, 95% CI= 0.083-1.584). The subjects living in rural areas and with low socioeconomic background may not be able to access or afford the appropriate oral hygiene aids and the health care facilities. Moreover, they may lack awareness about the importance of oral health and its proper care. This finding could be explained by the results from a study which revealed a higher percentage of children with good oral hygiene in upper socioeconomic group (9).

-Periodontal Status:

The overall mean number of healthy sextants in the study population was 2.32 ± 2.41 whereas in the children of comparable ages in Rajasthan state it was found to be 3.7 (25). The mean number of sextants affected by bleeding, calculus and shallow pockets were 1.31 ± 1.88, 2.33 ± 2.29 and 0.03 ± 0.18, respectively which is not in accordance to the findings by Jain M et al. (26) where mean number of sextants affected by periodontal disease were higher in the same age group. These differences may be due to reduced IQ level of children with mental retardation which hamper their oral health knowledge. The mean number of healthy sextants was significantly highest among the lower limb disability group (4.53 ± 2.06) stating the finest oral hygiene of the three groups studied. The mean number of sextants affected with bleeding, calculus and pockets were also significantly lowest among lower limb disabled group. The level of periodontal disease was even worse in the group with both upper and lower limb disability signifying their incapability in maintaining their own oral hygiene. Multivariate analysis elicited poor periodontal condition in upper limb disability group (OR= 2.382, 95% CI = 1.037-10.145) and in those with poor oral hygiene status (OR= 2.134, 95% CI = 1.695-26.498). This is in accordance with the findings of study by Karjalainen S et al. (27) where periodontal treatment was more common in subjects with physical inactivity. Moreover a high correlation between poor oral hygiene and the deve-lopment and progression of periodontal disease has been well documented and the role of poor oral hygiene as a risk factor of periodontal disease is well established (28).

The overall poor oral hygiene and periodontal status and a significant effect of limbs involved in disability on the oral hygiene and periodontal status of children who have Poliomyelitis urges for a system that assures a minimum level of human decency care for such children with adequate oversight of their continuing welfare. The highly alarming situation needs immediate attention as their dental health status is related to their social acceptability. A more extensive analysis of the differences in oral health based on physical inability of the subjects with disability is required to be carried out in various populations to provide a baseline data to develop better measures of disease and health, to explain the differences among population groups and to develop interventions targeted at eliminating morbidity.

Even though efforts have been made in the western world to improve the oral health of these less fortunate children, no attention has been directed by the health authorities in India. In our opinion, oral health care should be approached jointly with general health care in order to achieve a more holistic view of the individual’s physiological and psychological well-being.

## References

[1] Koneru A, Sigal MJ (2009). Access to Dental Care for Persons with Developmental Disabilities in Ontario. J Can Dent Assoc.

[2] (2000). Guidelines for oral health care for people with a physical disability.

[3] (2004). Guidelines for oral health care for people with a physical disability.

[4] (2010). New Delhi: Office of the Registrar General & Census Commissioner, India; c2010-11.

[5] (2010). Global Polio Eradication Initiative. The disease and the virus.

[6] Walton AG, Welbury RR, Thomsan JM, Foster HE (1999). Oral health and juvenile idiopathic arthritis: a review. Rheumatology.

[7] Pischon N, Pischon T, Kröger J, Gülmez E, Kleber BM, Bernimoulin JP (2008). Association among rheumatoid arthritis, oral hygiene, and periodontitis. J Periodontol.

[8] Chu CH, Lo EC (2010). Oral health status of Chinese teenagers with cerebral palsy. Community Dent Health.

[9] Oredugba FA, Akindayomi Y (2008). Oral health status and treatment needs of children and young adults attending a day centre for individuals with special health care needs. BMC Oral Health.

[10] Denloye OO (1998). Oral Hygiene status of mentally handicapped school children in Ibadan, Nigeria. Odontostomatol Trop.

[11] (2010). New Delhi: Office of the Registrar General & Census Commissioner, India; c2010-11.

[12] Agarwal A (2008). Social Classification: The need to update in the present scenario. Indian J Community Med.

[13] Greene JC, Vermillion JR (1964). The simplified oral hygiene index. J Am Dent Assoc.

[14] Purohit BM, Acharya S, Bhat M (2010). Oral health status and treatment needs of children attending special schools in South India: a comparative study. Spec Care Dentist.

[15] Sogi GM, Bhaskar DJ (2002). Dental caries and oral hygiene status of school children in Davangere related to their socio-economic levels: an epidemiological study. J Indian Soc Pedod Prev Dent.

[16] Sigal A (2009). Time to improve access to oral health care for persons with special needs. J Can Dent Assoc.

[17] Nahar SG, Hossain MA, Howlader MB, Ahmed A (2010). Oral health status of disabled children. Bangladesh Med Res Counc Bull.

[18] Pradhan A, Slade GD, Spencer AJ (2009). Access to dental care among adults with physical and intellectual disabilities: residence factors. Aust Dent J.

[19] Davis MJ (2009). Issues in access to oral health care for special care patients. Dent Clin North Am.

[20] Kane D, Mosca N, Zotti M, Schwalberg R (2008). Factors associated with access to dental care for children with special health care needs. J Am Dent Assoc.

[21] Brown JP, Schodel DR (1976). A review of controlled surveys of dental disease in handicapped persons. ASDC J Dent Child.

[22] Choi NK, Yang KH (2003). A study on the dental disease of the handicapped. J Dent Child.

[23] Gizani S, Declerck D, Vinckier F, Martens L, Marks L, Goffin G (1997). Oral health condition of 12- year-old handicapped children in Flanders (Belgium). Community Dent Oral Epidemiol.

[24] Mitsea AG, Karidis AG, Donta-Bakoyianni C, Spyropoulos ND (2001). Oral health status in Greek children and teenagers, with disabilities. J Clin Pediatr Dent.

[25] Bali RK, Mathur VB, Talwar PP, Chanana HB (2004). National Oral Health Survey and fluoride mapping..

[26] Jain M, Mathur A, Sawla L, Choudhary G, Kabra K, Duraiswamy P (2009). Oral health status of mentally disabled subjects in India. J Oral Sci.

[27] Karjalainen S, Vanhamäki M, Kanto D, Kössi L, Sewón L, Salo M (2002). Long-term physical inactivity and oral health in Finnish adults with intellectual disability. Acta Odontol Scand.

[28] Petersen PE, Ogawa H (2005). Strengthening the prevention of periodontal disease: the WHO approach. J Periodontol.

